# Academic procrastination among tunisian medical students: prevalence and associated factors

**DOI:** 10.1192/j.eurpsy.2023.1170

**Published:** 2023-07-19

**Authors:** F. Sahnoun, M. Turki, A. Guermazi, O. Elleuch, M. Ksibi, N. Halouani, S. Ellouze, J. Aloulou

**Affiliations:** Psychiatry B Department, Hedi Chaker University hospital, Sfax, Tunisia

## Abstract

**Introduction:**

Medical students have to do multiple tasks as part of their extensive curriculum in order to achieve the proficiencies expected of them. Being overwhelmed creates a time management problem, substance use and a tendency to procrastinate. Therefore, accumulated tasks may generate distress that could result in poor academic performance.

**Objectives:**

The aim of this study was to investigate prevalence and factors related to academic procrastination in Tunisian medical students.

**Methods:**

It was a cross-sectional, descriptive and analytical study conducted among Tunisian medical students. Data were collected through an anonymous online questionnaire, assessing sociodemographic characteristics, the “Tuckman Procrastination Scale” (TPS) and the “Time Management Subscale of the Learning and Study Strategies Inventory” (LASSI-TM).

**Results:**

A total of 133 participants completed the questionnaire. Their mean age was 26 ± 3,8 years, with a sex-ratio (F/M) of 4,5.

The mean LASSI-TM score was 16.69±4.6. Among students, 65.4 % showed deficit in time management.

The mean TPS score was 42.48±7.11. According to this scale, 87.2% of participants were engaged in academic procrastination. TPS score was significantly higher among psychoactive substances users (p=0.004), in those with psychiatric history (p=0.026) and in students with a rank over than 100 (p=0.029). It was negatively correlated with LASSI-TM score (p<0.001; r=-0.706).

**Conclusions:**

Considering the heavy load of work that the students undergo, it would be prudent to arrange for group trainings and workshops that will cultivate students with strategies and skills for effective time management, so that the tendency to procrastinate will be managed and their academic performance would improve.

**Disclosure of Interest:**

None Declared

